# Prevalence of depression in people living with HIV and AIDS at the Kalafong Provincial Tertiary Hospital Antiretroviral Clinic

**DOI:** 10.4102/sajpsychiatry.v25i0.1175

**Published:** 2019-07-22

**Authors:** Brett van Coppenhagen, Helene S. Duvenage

**Affiliations:** 1 Department of Family Medicine, University of Pretoria, Pretoria, South Africa

**Keywords:** AIDS, Antiretroviral Therapy, Depression, HAART, HIV, Kalafong, Prevalence

## Abstract

**Background:**

Compared to the general population, there is an increased prevalence of depression in people living with HIV and AIDS (PLWHA). The combination of these two common illnesses has profound consequences on the patient and on the healthcare system.

**Objective:**

This study determined the prevalence of depressive symptomatology in PLWHA attending the Kalafong Hospital ARV Clinic. The study also established if the patients received definitive treatment for unipolar depression.

**Methods:**

A cross-sectional, descriptive study was carried out on 622 adult patients, aged 18 years or older. A brief rating scale for depression, the Centre for Epidemiological Study Depression Scale (CES-D) was administered to participants. The CES-D is a 20-item self-rating scale that assesses current levels of depression as per DSM-IV criteria. The traditional score of 16 and above was used to define a case of depression.

**Results:**

The prevalence of depression according to CES-D scale was 53.8%. The study found that none of the 622 patients ever received definitive treatment for depression. A lower CD4 count is associated with more depressive symptomatology, most significantly in patients with a CD4 count of 50 or less.

**Conclusions:**

Depressive symptomatology was highly prevalent in the study patients. Despite the high prevalence, none of the study sample patients were treated for clinical depression. The findings reflect the importance of evaluating for depression in PLWHA, especially in high-risk groups such as patients presenting for their initiation visit or patients with a CD4 count of 50 or less. Depression remains under-recognised and under-treated in PLWHA.

## Introduction

Depression is a common mental disorder (CMD) that presents with low mood, loss of interest or pleasure, disturbed sleep or appetite, feelings of guilt or low self-worth, low energy and trouble concentrating. Depression is common, affecting more than 300 million people worldwide.^1^ Depression is the leading cause of disability worldwide and is a major contributor to the global burden of disease.^2^ At its worst, depression can lead to thoughts of death and suicide, with a confirmed 800 000 people taking their own lives each year globally.^1,3^ For every person who commits suicide, there are 20 more who make an attempt. Unlike clinically obvious mental illness like psychosis, depression remains hidden, not treated or talked about.

International and South African studies have shown a wide variation in the prevalence of depression, but overall this prevalence is approximately 10% in general populations.^4,5,6^ The evidence highlights a nearly twofold increased prevalence of depression in people living with HIV and AIDS (PLWHA), compared to the general population.^7,8^ The combination of these two common illnesses has profound consequences because of a substantial impact on the person living with HIV and AIDS and, in the larger picture, on our healthcare system.

The combination of HIV and depression has been shown to lower the likelihood of receiving antiretroviral treatment (ART), worsen ART adherence and result in higher viral loads with a decline in CD4 cell count. Comorbid HIV and depression leads to a faster progression to AIDS and the development of AIDS-defining clinical conditions, increased physical symptoms and a worsening quality of life. High-risk behaviours, including unsafe sexual practice and the increased risk for excessive alcohol consumption and illicit drug use, become more prevalent. The depression and HIV combination results in an increased mortality in PLWHA.^8,9,10,11^

To complicate matters further, depressive symptoms can also be caused by many medications used in PLWHA, including common medication (metoclopramide, co-trimoxazole, corticosteroids, benzodiazepines, etc.), tuberculosis treatment (INH, etc.) or even the antiretrovirals (ARVs) themselves. Most of the antiretroviral agents have neuropsychiatric side effects. The most common neuropsychiatric side effects of ARVs are insomnia and headache. Efavirenz is the agent most commonly implicated with the acute onset of depression and suicidality. Lamivudine, stavudine and tenofovir all have a potential for depression as a side effect.^12,13^ Withdrawal of the offending drug often leads to resolution of the depression.

Olley et al.^14^ found that 34.9% of their sample met the diagnostic criteria for major depressive disorder at the South African ARV Clinic of Tygerberg Hospital, Cape Town. Freeman et al.^15^ found that the prevalence of major depressive disorder in PLWHA in South African clinics was 11.1% and 29.9% for mild depression.

Tsai,^16^ who did a meta-analysis of 11 studies with 4461 PLWHA in sub-Saharan Africa, found the pooled prevalence of major depressive disorder to be 14.5%.

Depression and depressive disorders are the most common psychiatric illness in PLWHA.^15^ Compliant antidepressant treatment is associated with improved HAART initiation and adherence.^17,18,19^

Unfortunately, depression remains under-diagnosed and under-treated in PLWHA.^20^ The South African Department of Health’s National Antiretroviral Treatment Guidelines of 2004 stipulated that as part of patient selection criteria for ART initiation, patients should have ‘no untreated active depression’ or ‘no active alcohol or other substance abuse’.^21^ The 2010 guidelines also recommend doing a psychosocial assessment or mental health screen at the first visit to promote adherence.^22^

In South Africa, it is thus still a requirement that all PLWHA receive some form of evaluation for depression and substance abuse prior to initiation of ARVs. Unfortunately, with the growing burden of mental health disorders, especially in PLWHA, there remains a considerable mental health treatment gap in South Africa.^23^

The under-recognition and under-treatment of depression in PLWHA is in part because of the complex and confounding overlap between the somatic symptoms of HIV itself, its comorbid illnesses and the neurovegetative symptoms of depression. Signs of depression such as disturbed sleep, loss of appetite, weight loss, fatigue, poor concentration and memory can also be because of the HIV infection itself, making diagnosis more difficult. The DSM-IV or DSM-V does not have specific criteria for the recognition of depression in the ill or in PLWHA.^12,24^ This leaves the clinician to use best judgement without guide. It is easy for the clinician to attribute the major depressive disorder’s symptoms to the HIV infection or comorbid illness, thus overlooking the depression. An incorrect judgement on the clinician’s part would be that the patient’s depression will improve on ARVs alone.

This research primarily aimed to determine the prevalence of depression symptomatology in PLWHA at the Kalafong Provincial Tertiary Hospital ARV Clinic. The secondary aim was to see if there was an improvement in depression symptomatology after 1 year of treatment with ARVs. The researchers also wanted to know if the patients received treatment for clinical depression.

## Methods

A cross-sectional, descriptive study was carried out on 622 adult patients, aged 18 years or older, over an 8-month period in 2012 at the Kalafong Hospital ARV Clinic.

Kalafong Hospital is a tertiary hospital situated in the western region of Tshwane, Pretoria, and receives referrals from surrounding general practitioners and primary healthcare centres. Mental health services at Kalafong Hospital are restricted to inpatient 72 h observation, as per *Mental Health Care Act*, with outpatient psychiatric and psychologist services offered only at the local primary healthcare centres. The integration of mental healthcare is limited to the attentiveness of the ARV Clinic doctors, nurses, affiliated social workers and lay counsellors.

All patients from two groups were invited to participate in the study. A First Visit Group included all first visit patients, pre-initiation of ARVs. A One Year Plus Group included all patients 1 year or more post-initiation of ARVs. The study was conducted over an 8-month period from January 2012 to August 2012. All patients that attended the clinic had confirmed initial CD4 of 200 or less, as per the national guideline of providing ARVs at this period in time.

**FIGURE 1:**
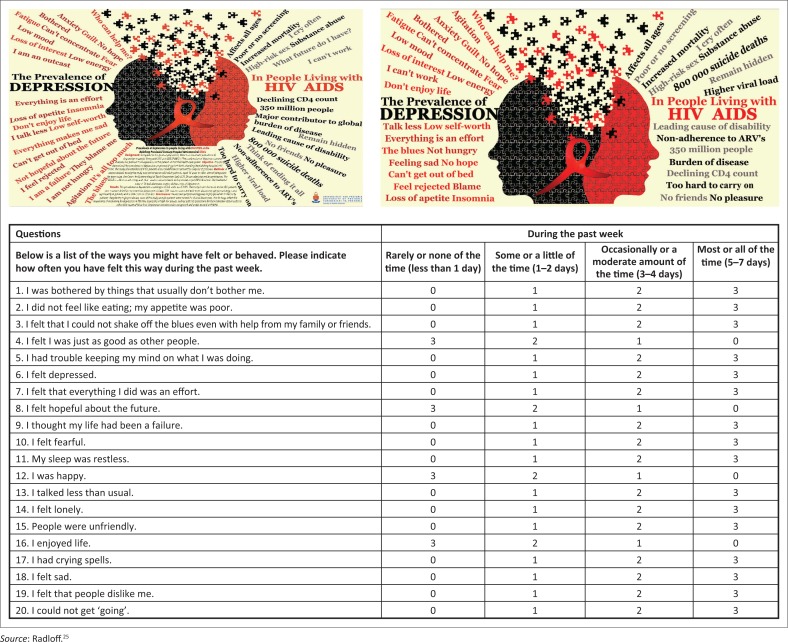
Centre for Epidemiological Studies Depression Scale (CES-D) scoring: 0 for answers in the first column, 1 for answers in the second column, 2 for answers in the third column, 3 for answers in the fourth column. The scoring of positive items is reversed, that is, items 4, 8, 12 and 16. Possible range of scores is 0–60, with the higher scores indicating the presence of more symptomatology.

A brief rating scale for depression, the Centre for Epidemiological Study Depression Scale (CES-D) was administered to participants (Figure 1).^25^ The CES-D was selected for this study based on the scale’s brevity, disorder/depression specificity, ease of administration and existing evidence for cross-cultural administration. The CES-D is a 20-item self-rating scale that assesses current levels of depression as per DSM-IV criteria and has been validated in many international general population samples including South Africa.^26,27^ The CES-D has also been validated specifically in PLWHA with a demonstrated sensitivity of 79% (95% CI: 76% – 83%) and a specificity of 61% (95% CI: 56% – 85%).^26^ The traditional score of 16 and above was used to define a case of likely depression.

A research assistant, who participated for the duration of the study, was an experienced lay counsellor who did pretest and post-test HIV as well as ARV counselling at the Kalafong Hospital voluntary HIV counselling and testing (VCT) and ARV Clinic. The research assistant was competent in several languages including English, Setswana, IsiZulu and basic Afrikaans, and verbally administered the CES-D. Care was taken with forward and backward translation process of the CES-D research questionnaire to and from English, Setswana and Afrikaans. Prior to study initiation or data collection, the assistant was trained in research ethics in order to ensure effective communication, confidentiality, informed consent and impartiality.

Patients who qualified as a case of depression were issued with a verbal explanation reports and referred to the Kalafong ARV Clinic doctor and to the nearest mental health clinic for formal psychological and psychiatric assessment. Even though the CES-D screening tool does not enquire about suicidality, participants of the study who volunteered or expressed suicidal ideation, intent or plan were facilitated by the interviewer to the Kalafong ARV Clinic doctor in person for further evaluation and management.

Patient participation was entirely voluntary and patients were not reimbursed, paid, influenced or coerced into participation. The study was implemented after the approval from the University of Pretoria Faculty of Health Sciences Research Ethics Committee. The Biostatistics Unit of the South African Medical Research Council in Pretoria performed the data analysis.

Data analysis was performed with the use of Stata release 11 statistical software and prevalences were expressed as percentages and reported along with 95% confidence intervals. Prevalences for the ARV naïve First Visit Group versus ARV One Year Plus Group were compared with Pearson’s chi-square test, and a 95% confidence interval was computed for the difference in prevalence between the groups.

## Ethical consideration

Ethical approval to conduct the study was obtained from the University of Pretoria Faculty of Health Sciences Research Ethics Committee (reference no. 137/2011).

## Results

### Sample characteristics

The study included a total sample size of 622 patients diagnosed with AIDS, with a mean age of 38.9 years. The ages ranged from 19 to 68 years. The majority (61.4%) of the total study sample was under the age of 41. The age group with the majority (44.6%) of the patients was in the 31- to 40-year age group. Female patients made up the majority of the sample (63.5%). Age categories were relatively equally represented in gender, except for the 18- to 30-year age group, which consisted of more female patients (13.99% of the total number of patients) compared to male patients (2.73% of the total number of patients). All patients of the study sample were black or African descent. The majority of patients could speak English 60.0% followed by IsiZulu 25.0% and Setswana 18.0% (Tables 1 and 2).

**TABLE 1:** The characteristics of study sample.

Socio-demographic variables	Number of patients or mean		Percentage of total or standard deviation	
	*n*	mean	%	s.d.
**Total sample**	622	-	100	-
**Gender**				
Male	227	-	36.5	-
Female	395	-	63.5	-
**Age (range min 19 to max 68)**				
Total	-	38.9	-	8.72
Male	-	41.4	-	8.39
Female	-	37.4	-	8.60
**Age categories**				
18–30 years	104	-	16.7	-
31–40 years	278	-	44.6	-
41–50 years	182	-	29.2	-
51–60 years	42	-	6.7	-
Over 60 years	16	-	2.5	-
**Race groups**				
African	622	-	100	-
Other race groups	0	-	0	-
**Language proficiency**				
English	376 of 622	-	60.4	-
Zulu	158 of 622	-	25.4	-
Tshwana	114 of 622	-	18.3	-
Afrikaans	29 of 622	-	4.6	-

*n*, number; s.d., standard deviation.

**TABLE 2:** Sample characteristics with gender ratios and mean ages.

Age categories	Female		Male		Total	
	*n*	%	*n*	%	*n*	%
18–30 years	87	13.99	17	2.73	104	16.72
31–40 years	179	28.78	99	15.92	278	44.69
41–50 years	100	16.08	82	13.18	182	29.26
51–60 years	21	3.38	21	3.38	42	6.75
Over 60 years	8	1.29	8	1.29	16	2.57
**Total range 19–68 years**	**395**	**63.5**	**227**	**36.5**	**622**	**100.0**

### Prevalence of cases of depression at the Kalafong ARV Clinic

The total prevalence of cases of depression as per screening was 53.8%. No statistical difference was found (*p* = 0.129) in the prevalence of depressive symptomatology between females (55.7%) and males (50.6%) (Table 3).

**TABLE 3:** Prevalence of cases of depression with gender ratios.

Prevalence of cases of depression	Female		Male		Total		*p*
	*n*	%	*n*	%	*n*	%	
Cases without depression	175	44.30	112	49.34	287	46.14	0.243
Cases of depression	220	55.70	115	50.66	335	53.86	0.129
**Total**	**395**		**227**		**622**		**-**

The study found that none of the 622 sample patients received definitive treatment for clinical depression.

There was a significant difference in the prevalence of cases of depression in the First Visit Group (who were ARV naive) 60.84% as compared to the group of patients that are 1 year post-initiation of ARV therapy 51.77% (*p* = 0.035) (Table 4).

**TABLE 4:** Prevalence of cases of depression in First Visit Group compared to the One Year Plus Group.

Variable	Total sample			First Visit			One Year Plus		
	*n*	%	s.d.	Group			Group		
				*n*	%	s.d.	*n*	%	s.d.
Number of patients	622	-	-	143	-	-	479	-	-
Percentage of total	-	100	-	-	23	-	-	77	-
CD4 mean	255	-	179	87	-	77	305	-	170
Cases of depression number	335	-	-	87	-	-	248	-	-
Percentage	-	53.8	-	-	60.84	-	-	51.7	-

*n*, number; s.d., standard deviation.

### Additional findings

Patients with a lower CD4 count demonstrated higher levels of depressive symptomatology, most significantly in patients with a CD4 count of 50 or less (Table 5).

**TABLE 5:** Relationship between CD4 count and cases of depression.

CD4 category	Total number patients	Cases of depression in category	Prevalence depression cases (%)	Odds ratios	95% confidence intervals
≤ 50	74	53	71	1.0	reference
≥ 50 – ≤ 100	49	26	53	0.447	0.206–0.969
≥ 100 – ≤ 150	73	45	61	0.636	0.316–1.279
≥ 150 – < 200	81	40	49	0.386	0.194–0.768
≥ 200	345	171	49	0.389	0.223–0.679
**Total**	**622**	**335**	**53.86**	**-**	

## Discussion

The study includes a significant total sample size of 622 patients of which the majority (63.0%) was female. This higher percentage of female patients corresponds with the usual gender ratio at the Kalafong ARV Clinic, where two-thirds of the patients are female. This finding is congruent with the fact that, in sub-Saharan Africa, the HIV and AIDS epidemic shows the highest prevalence among women.^9^

The ages of the participants in the study ranged from 19 to 68 years with a mean age of 38 years. The mean age of the male patients (41 years) was 4 years older than that of the female patients (37 years). This is consistent with the finding that sub-Saharan African women acquire HIV infection at least 5–7 years earlier than men.^28^ Age categories are relatively equally represented by genders, except for in the 18- to 30-year age group. This group consists of more female patients (13.99% of total patients) compared to male patients (2.73% of total patients). These results are consistent with the UNAIDS GAP report of 2012 stating that young women in the age group of 15–24 years old in sub-Saharan Africa are twice as likely as young men of the same age to be living with HIV.^28^

### Prevalence of depressive symptomatology

This study found the total prevalence of cases with depression as per CES-D screening to be 53%. In comparison, Tsai^16^ found the prevalence of probable depression among persons with HIV in sub-Saharan Africa to be 30.2%. This prevalence demonstrates that depressive symptomatology was higher at the Kalafong ARV Clinic than the average for the rest of sub-Saharan Africa, as per Tsai.^16^ The pooled prevalence of 30.2% was from 11 studies with 4461 participants. In these studies, the CES-D was the most frequently used instrument. Even with a relatively high rate of false-positive cases identified through screening, the Tsai meta-analysis confirmed a high 14.5% prevalence of major depressive disorder.

Despite the prevalence of depressive symptomatology being marginally more in females than males, no statistical difference was found (*p* = 0.129) in the prevalence of depressive symptomatology between the female study group (55%) and the male study group (50%). Worldwide it has been found that in the general population, female gender is a risk factor for depression and two to three times more women experience depression than men.^29,30^ In PLWHA, Freeman et al.^15^ found no difference between men and women in their levels of depression or prevalence of mood disorder. The point was argued to explain possible equality of depression prevalence in PLWHA; however, it was conceded that more research was required to explain this uncharacteristic finding. In opposition, Shittu et al.^31^ found a higher prevalence of depression in women although they also summarised that studies worldwide found that the prevalence of depression varied considerably based on the gender of PLWHA.

The study found that none of the 622 study patients were ever treated for major depressive disorder. This reflects on the inadequate recognition and treatment of major depressive disorder at a primary healthcare level, prior to referral for ARVs, at initiation or on follow-up of the patients at Kalafong ARV Clinic. This study indicates clearly that major depressive disorder is under-recognised and under-treated in this sample of PLWHA.

In spite of not receiving treatment for depression, there was a significant decrease in the prevalence of cases of depression in the First Visit ARV Naive Group (60.84%) compared to the group of patients that are 1 year post-initiation of ARV therapy (51.77%). One year of intervention with ARV therapy appears to improve depressive symptomatology in PLWHA.

The fact that none of the sample patients were ever treated for depression imparted significance to the decrease in depressive symptomatology after one year of ARV therapy. If any of the sample patients had been treated for depression, then the relationship between depressive symptomatology and duration on ARVs could not be attributed to the treatment intervention of ARV therapy.

The improvement of depressive symptomatology may thus possibly be attributed to the first year of ARV therapy with subsequent lowering of viral load, improvement of CD4, resolution of opportunistic infections and improvement of life quality.

Despite this improvement in depressive symptomatology after the first year on ARVs, the prevalence remains high and the evaluation for depression is still indicated, even after the first year on ARVs.

Patients with a lower CD4 count were associated with more depressive symptomatology. It was most significant in patients with a CD4 count of 50 or less (71%). The First Visit Group of patients had the second highest prevalence of depressive symptomatology (60%). These two groups (patients visiting for the first time or patients with a CD4 of 50 or less) can thus be classified as high-risk groups and should be prioritised for evaluation of clinical depression.

### Limitations

This research is limited to patients who attended the Kalafong Hospital ARV Clinic and to patients who were able to complete the research questionnaire comprehensively. Patients with severe cognitive impairment, severe medical illness and severe mental illness were excluded. The prevalence of depression was determined by screening only. No formal evaluations for depression were conducted as part of the study. Viral loads were not measured or considered as part of this study. Other confounding variables like social circumstances, additional treatment or comorbid illnesses which may have an effect on depressive symptomatology were not considered.

## Conclusion

Depressive symptomatology was highly prevalent in the study patients. Despite the high prevalence of depressive symptomatology, surprisingly none of the study sample patients were ever treated for major depressive disorder. This fact points towards inadequate evaluation and treatment of depression in PLWHA, not only at Kalafong ARV Clinic but also at the primary healthcare referral centres. Clinical depression remains under-recognised and under-treated in this sample of PLWHA.

The study results suggest that patients who have been on ARV therapy for longer than a year showed an improvement in depressive symptomatology despite not receiving treatment for clinical depression. Further research in this field is recommended.

The study identifies the need for evaluating for depression in PLWHA, particularly in high-risk groups such as patients at their first or initiation visit or patients with a CD4 count of 50 or less.

The combination of these illnesses has profound consequences and a substantial impact, not only on the individual living with HIV and AIDS but also on our healthcare system. As growing numbers of PLWHA are enrolled into ARV programmes, the responsibility falls on the healthcare team to adequately and aggressively detect and treat depression in this high-risk group of patients.
